# Lipidomics of Glycosphingolipids

**DOI:** 10.3390/metabo2010134

**Published:** 2012-02-02

**Authors:** Hany Farwanah, Thomas Kolter

**Affiliations:** Life and Medical Sciences Institute (LiMES), Membrane Biology and Lipid Biochemistry Unit, c/o Kekulé-Institut für Organische Chemie und Biochemie, University of Bonn, Gerhard-Domagk Str. 1, D-53121 Bonn, Germany; Email: hany.farwanah@uni-bonn.de

**Keywords:** glycolipids, glycomics, lipidomics, mass spectrometry, sphingolipids

## Abstract

Glycosphingolipids (GSLs) contain one or more sugars that are attached to a sphingolipid moiety, usually to a ceramide, but in rare cases also to a sphingoid base. A large structural heterogeneity results from differences in number, identity, linkage, and anomeric configuration of the carbohydrate residues, and also from structural differences within the hydrophobic part. GSLs form complex cell-type specific patterns, which change with the species, the cellular differentiation state, viral transformation, ontogenesis, and oncogenesis. Although GSL structures can be assigned to only a few series with a common carbohydrate core, their structural variety and the complex pattern are challenges for their elucidation and quantification by mass spectrometric techniques. We present a general overview of the application of lipidomics for GSL determination. This includes analytical procedures and instrumentation together with recent correlations of GSL molecular species with human diseases. Difficulties such as the structural complexity and the lack of standard substances for complex GSLs are discussed.

## Abbreviations

GSL nomenclature: [1]
Ac: Acetyl
APCI: Atmospheric Pressure Chemical Ionization
APPI: Atmospheric Pressure Photo-Ionization
Cer: Ceramide (*N*-Acylsphingosine)
CID: Collision induced dissociation
DESI: Desorption electrospray ionization
ESI: Electrospray ionization
Forssman-GSL: GalNAcα1,3GalNAcβ1,3Galα1,4Galβ1,4Glcβ1,1′Cer
FT-ICR: Fourier-Transform-Ion-Cyclotron
GA1: Galβ1,3GalNAcβ1,4Galβ1,4Glcβ1,1′Cer
GA2: GalNAcβ1,4Galβ1,4Glcβ1,1′Cer
Gal: D-Galactose
GalCer: β-Galactosylceramide
GalNAc: 2-Deoxy-2-*N*-Acetyl-D-Galactosamine
Gb3Cer: Galα1,4Galβ1,4Glcβ1,1′Cer
Gb4Cer: GalNAcβ1,3Galα1,4Galβ1,4Glcβ1,1′Cer
GD1a: Neu5Acα2,3Galβ1,3GalNAcβ1,4( Neu5Acα2,3)Galβ1,4Glcβ1,1′Cer
GD1b: Galβ1,3GalNAcβ1,4( Neu5Acα2,8 Neu5Acα2,3)Galβ1,4Glcβ1,1′Cer
GD2: GalNAcβ1,4( Neu5Acα2,8 Neu5Acα2,3)Galβ1,4Glcβ1,1′Cer
GD3: Neu5Acα2,8 Neu5Acα2,3Galβ1,4Glcβ1,1′Cer
Glc: D-Glucose
GlcNAc: 2-Deoxy-2-*N*-Acetyl-D-Glucosamine
GM1(a): Galβ1,3GalNAcβ1,4(NeuAcα2,3)Galβ1,4Glcβ1,1′Cer
GM1b: Neu5Acα2,3Galβ1,3GalNAcβ1,4Galβ1,4Glcβ1,1′Cer
GM3: Neu5Acα2,3Galβ1,4Glcβ1,1′Cer
GQ1b: Neu5Acα2,8Neu5Acα2,3Galβ1,3GalNAcβ1,4(Neu5Acα2,8Neu5Acα2,3)Galβ1,4Glcβ1,1′Cer
GSL: Glycosphingolipid
GT1a: Neu5Acα2,8 Neu5Acα2,3Galβ1,3GalNAcβ1,4( Neu5Acα2,3)Galβ1,4Glcβ1,1′Cer
GT1b: Neu5Acα2,3Galβ1,3GalNAcβ1,4( Neu5Acα2,8 Neu5Acα2,3)Galβ1,4Glcβ1,1′Cer
HDL: High Density Lipoprotein
HexCer: Hexosylceramide (GlcCer or GalCer)
HexNAc: *N*-Acetylhexosamine (usually GlcNAc or GalNAc)
HILIC: Hydrophilic interaction liquid chromatography
IR: Infrared
LacCer: Lactosylceramide
LC: Liquid chromatography
LDL: Low Density Lipoprotein
LTQ: Linear trap quadrupole
lysoGSL: glycosylated sphingoid bases
MDCK: Madin Darby canine kidney
MS/MS: Tandem mass spectrometry
MRM: Multiple reaction monitoring
MS: Mass spectrometry
*m/z*: Mass per charge ratio
Neu5Ac: *N*-Acetylneuraminic Acid
Neu5Gc: *N*-Glycolylneuraminic Acid
QTOF: Quadrupole Time-of-Flight
RP: Reversed phase
Sulfatide: GalCer-3-sulfate
MALDI: Matrix-Assisted Laser Desorption/Ionization
TLC: thin layer chromatography
TOF: Time-of-Flight
VLDL: Very Low Density Lipoprotein

In the mentioned GSLs, the hexoses are present as pyranosyl forms

## 1. Introduction

Within systems biology, lipidomics is the discipline that creates, analyzes, and integrates complex data on the lipidome of so-called systems. These can be cells, tissues, organs, biofluids, or whole organisms. Ideally, lipid profiles can be correlated with data about genome, transcriptome, proteome, and other metabolites of a system. Lipidomics as a technological platform relies largely on mass spectrometry (MS) as the key analytical technique. Lipids comprise a structurally very heterogeneous group of metabolites that can differ drastically in abundance and physical and functional properties. A noteworthy heterogenous and difficult to determine group of lipids are the glycolipids. Glycolipids are essential components of the surfaces of living cells [2]. Within the comprehensive classification system suggested by the LIPID MAPS consortium, glycosylated lipids are classified according to the identity of their core lipids, and also as saccharolipids when fatty acyl groups are linked directly to a sugar backbone [3]. One major group of glycolipids is that of the glycosphingolipids (GSLs), which is further classified into acidic, basic, and amphoteric GSLs. Despite their various roles in human health and disease, GSL analysis is often neglected or incompletely addressed within “omics” approaches, even in lipidomics [4,5] and glycomics. This is mainly due to the structural complexity and diversity of cellular glycolipids, and also to differences in abundance, chemical stability, and biophysical properties between the analytes. The glycome represents the largest class of post-translationally modified biomolecules [6], and glycomics techniques [7] such as lectin [8] and glycan microarrays [9] have been only occasionally applied to glycolipids. Also most of the mass spectrometric techniques developed for glycan analysis are not directly applicable to these glycoconjugates [10]. In higher animals, most glycolipids are GSLs, in which the carbohydrate part is attached to a ceramide (*N*-acylsphingoid) moiety [2].

**Figure 1:**
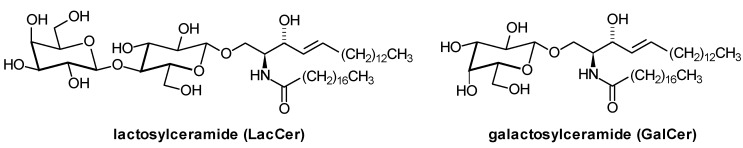
Structures of lactosylceramide and galactosylceramide. Only one of the various lipoforms is shown.

The glycan part can contain more than ten monosaccharide units that can differ in type, linkage, and additional modifications. As in the biosynthesis of other glycans, which usually occurs with a mixture of glycoforms, GSL structures are diverse since they arise from a combinatorial biosynthetic pathway [11]. Despite this great structural variety in the glycan part, evolutionary related organisms express only a limited set of so-called GSL series that share common carbohydrate sequences (Table 1). In higher animals, most series are derived from lactosylceramide (LacCer; Figure 1), in which lactose is β-glycosidically linked to the 1-hydroxyl group of ceramides. An exception is the gala series, which is derived from β-galactosylceramide (GalCer, Figure 1).

**Table 1:** Glycosphingolipid (GSL) series of higher eukaryotes [1].

Series	Core structure
Gala	Galα1,4Galβ1,1′Cer
Ganglio	Galβ1,3GalNAcβ1,4Galβ1,4Glcβ1,1′Cer
Globo	GalNAcβ1,3Galα1,4Galβ1,4Glcβ1,1′Cer
Isoglobo	GalNAcβ1,3Galα1,3Galβ1,4Glcβ1,1′Cer
Lacto	Galβ1,3GlcNAcβ1,3Galβ1,4Glcβ1,1′Cer
Neolacto	Galβ1,4GlcNAcβ1,3Galβ1,4Glcβ1,1′Cer
Muco	Galβ1,3Galβ1,4Galβ1,4Glcβ1,1′Cer

In addition to the species dependence, GSLs form cell-type specific glycan patterns that change with cell growth, differentiation, viral transformation, ontogenesis and oncogenesis. GSL heterogeneity results not only from variations within the carbohydrate part, but also within the lipid part, since the biosynthetic machinery that generates the lipid moiety gives rise to “lipoforms” [12]. This can in part be attributed to the different acyltransferases encoded by the lass-genes [13] that incorporate the amide-bound fatty acids and differ in their specificity towards the acyl coenzyme A thioesters of different chain lengths. An example is GSLs with polyunsaturated acyl chains that are required for male fertility [14]. Also sphingoid bases found in GSLs differ in chain length, and in the degree of desaturation and hydroxylation.

**Figure 2:**
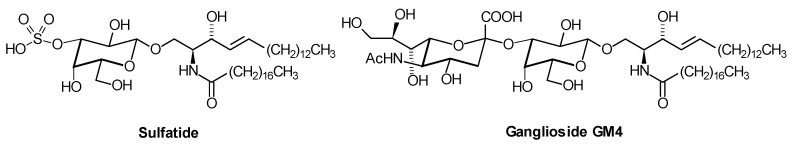
Structures of sulfatide (GalCer-3′-sulfate) and ganglioside GM4. Only one of the various lipoforms is shown. Sulfatide is the major storage substance in Metachromatic Leukodystrophy.

**Figure 3:**
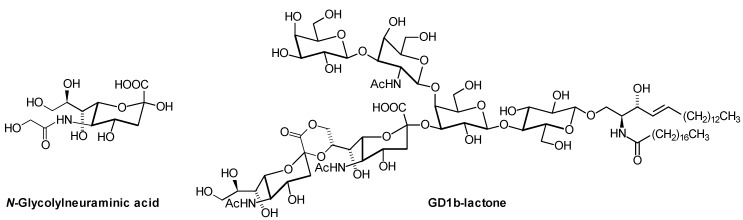
*N*-Glycolylneuraminic acid (Neu5Gc; α-configuration) and an example of a GD1b-lactone.

With the exception of lysoglycosphingolipids (lysoGSLs; glycosylated sphingoid bases), most GSLs are electrically neutral or are negatively charged. The most abundant negatively charged GSLs are the gangliosides and the sulfoglycosphingolipids (sulfatides, Figure 2). Gangliosides are sialic acid-containing GSLs. Sialic acids are derivatives of neuraminic acid, with *N*-acetylneuraminic acid (Neu5Ac) as the most important one. Sialic acids can be attached to several galactosyl residues within the core structures of the ganglio-, lacto-, and neolacto-series, but also to GalCer (ganglioside GM4; Figure 2). They can be further modified by acylation of different alcohol groups [15], or by lactonisation [16] (Figure 3). These modifications can be lost during GSL isolation and workup under alkaline conditions, which are often applied to remove glycerophospholipids that contain fatty acids in ester-linkage [17]. Although removal of other lipid classes can reduce signal suppression and improve the sensitivity of GSL determination, this alkaline treatment is not mandatory. For example, chloroform/methanol extraction in a ratio of 1:1 was used to analyze polar lipids including phospholipids and GSLs from glioblastoma cells by nano-LC FT-ICR MS and quadrupole linear ion trap MS/MS [18]. *O*-acetylated gangliosides from tissues can be determined after chloroform/methanol extraction in a ratio of 1:2 and a subsequent partition step, as applied to human gliosarcoma [19]. *O*-Acetylated sialic acids in gangliosides occur especially in growing cells and tissues and are regarded as oncofetal markers present on different tumors [20]. As part of glycolipids and glycoproteins, they serve as receptors for coronaviruses [21]. Another modified sialic acid is *N*-glycolylneuraminic acid (Neu5Gc, Figure 3). Neu5Gc as component of glycoconjugates is known as the so-called Hanganutziu-Deicher antigen, which is found in human tissues only in trace amounts, with the exception of certain tumors and in fetuses. Determination of Neu5Gc and Neu5Ac-containing gangliosides is either achieved by classical chromatographic techniques, or, with higher sensitivity, by hyphenation of chromatography with ESI-MS [22]. As in most other glycoconjugates, sialic acids occur only in the α-anomeric configuration.

**Figure 4:**
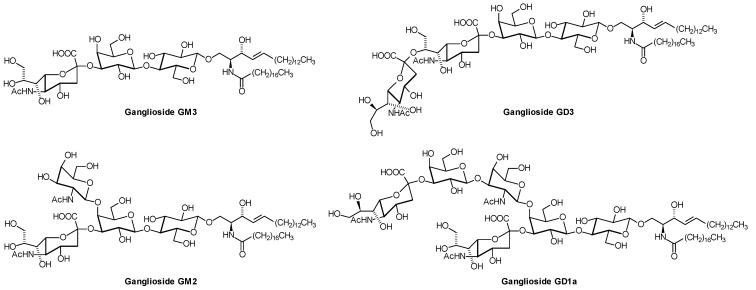
Structures of gangliosides GM3, GM2, GD3, and GD1a. Only one of the various lipoforms is shown. GD1a is the most abundant ganglioside in the adult human brain. Ganglioside GM2 is the major storage substance in Tay-Sachs disease (β-hexosaminidase α-subunit deficiency), Sandhoff disease (β-hexosaminidase β-subunit deficiency), and GM2-activator deficiency.

**Figure 5:**
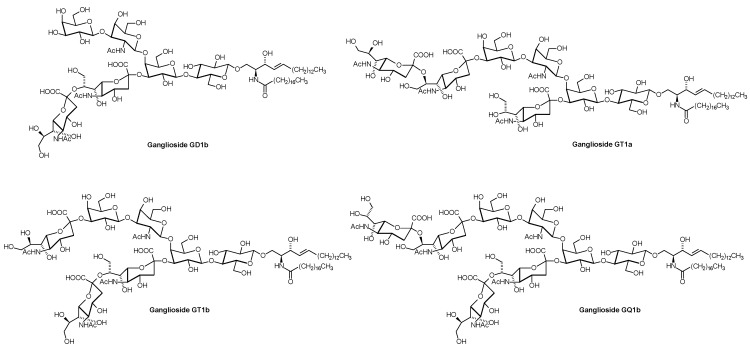
Structures of Oligosialogangliosides GD1b, GT1a, GT1b, and GQ1b. Only one of the various lipoforms is shown. Autoantibodies against ganglioside GM1 or different other gangliosides or combinations of them are the cause of Guillain-Barré- and Miller-Fisher syndrome [34].

For the nomenclature of gangliosides, the system introduced by Svennerholm [23] is used, which has been approved by IUPAC [1]. In brief, the name contains information about the series (G = ganglio), the number of neutral sugars (5-n), and number ("A" = 0, "M" = 1, "D" = 2, *etc.*) and position (a,b,c) of sialic acid residues. Gangliosides of the ganglio-series are especially found on neuronal cells. In the nervous system, they contribute to 10-12% of the lipid content [24]. During brain development, the ganglioside pattern changes from simple gangliosides like GM3 and GD3 (Figure 4) to more complex ones like GD1a (Figure 4) and GT1b (Figure 5) [25]. The function of the highly diverse GSL pattern on cellular surfaces is not clear, but glycan-protein interactions certainly play a role [26]. For example, the myelin-associated glycoprotein on oligodendrocytes recognizes Neu5Acα2,3Galβ1,3GalNAc-termini [27], so that the above mentioned change in ganglioside composition during brain development can be functionally correlated with myelination. Dependent on the deficient glycosyltransferase, genetically engineered mice with defects in ganglioside biosynthesis show defects in neural function, reproductivity, or insulin signalling [11]. Not only interactions between GSL glycans and lectins, but also carbohydrate-carbohydrate interactions are discussed [26].

**Figure 6:**
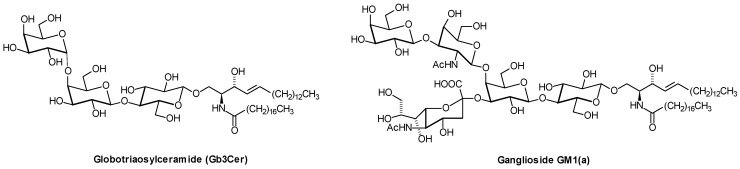
Structures of the toxin receptors Gb3Cer and ganglioside GM1. Only one of the various lipoforms is shown. Gb3Cer is the major storage substance in Fabry disease (α-galactosidase A deficiency), and GM1 in GM1-gangliosidosis (GM1-β-galactosidase deficiency).

Since GSLs also interact with proteins present in the same membrane [28], including pathophysiologically relevant receptors such as that for insulin [29], methods for their determination in different biological matrices are desired in relation to associated diseases. In addition, it is assumed that GSLs are not homogeneously distributed on the cell surface, but, together with glycosylphosphatidylinositol-anchored proteins, sphingomyelin, and cholesterol, segregate into membrane domains [28]. These so-called rafts are regarded as the physiological surroundings of many membrane proteins. According to Stimulated Emission Depletion microscopy of living cells, the lifetime of rafts appears to be very short, and also the upper limit for their size seems to be only about 20 nm diameter [30]. Although methods, such as extraction by means of detergent or immunostaining used for the characterization of these transient structures, have to be critically examined [31], analysis of lateral GSL distribution should also be a focus of glycosphingolipidomics. In addition, the subcellular distribution of GSLs, which is not resolved by most of the currently applied analytical techniques, is of relevance because GSL function can depend on the cellular site: Different functional properties have been attributed to ganglioside GD3, dependent on its localization in the plasma membrane, the mitochondria, or the nucleus [32]. In the plasma membrane, GSLs play a role in infectious diseases [33] where they act as cellular receptors and co-receptors for viruses, bacteria, and microbial toxins. Examples include ganglioside GM1 as the receptor for cholera toxin, and globotriaosylceramide (Gb3Cer) as receptor for verotoxins and Shiga toxins (Figure 6).

As revealed by classical analytical techniques, epithelial cells of the human intestine contain monoglycosylceramides (cerebrosides) and blood group-GSLs as major compounds together with smaller amounts of di-, tri-, and tetra-glycosylceramides and more complex GSLs [35]. Gb3Cer is apparently absent from these cells, and it is still an open question how Shiga toxins produced by *E. coli* strains in the intestine as the cause of hemorrhagic colitis and hemolytic uremic syndrome pass this barrier to enter the circulation [36]. On lipoproteins, the globo-series Shiga toxin receptors have been found especially on VLDL and LDL, as determined by ESI-MS [37]. Also gangliosides, mostly GM3, GD3, GD1a, GM2 (Figure 4), GT1b, sialylneolactotetraosylceramide, GD1b, and GQ1b (Figure 5), are also present in serum, where about 98% of them are transported by serum lipoproteins, predominantly by LDL (66%), followed by HDL (25%) and VLDL (7%) [38]. Very little is known about how this part of the lipidome changes with disease states and how it influences human health.

## 2. Glycosphingolipid Structure Elucidation and Analysis

Mass spectrometry has revolutionized the analysis of (glyco)lipids and allowed the emergence of the field of “lipidomics”, which aims at a comprehensive analysis of the (glyco)lipidome in a given system. In particular, it was the introduction of “new” ionization methods (ESI, APCI, MALDI, APPI, and nano-ESI), as well as the development of different analyzer types (triple quadrupoles, the different ion trap types, and QTOFs), which enabled a more rapid or a more comprehensive (glyco)lipid analysis even from crude lipid extracts without the need for prior separation (shotgun lipidomics). The fact that the new ionization methods are run under atmospheric pressure facilitated the laborious work carried out in older mass spectrometric ionization techniques, and allowed coupling with different chromatographic systems (TLC and liquid chromatography). On the other hand, the various scanning modes (product ion scan, neutral loss, and multiple reaction monitoring) in combination with high mass-resolution and -accuracy, which are achievable by the commercially available analyzers, enabled effective structural elucidation, profiling, and quantification of at least some (glyco)lipid classes of interest in reasonable periods of time.

**Figure 7:**
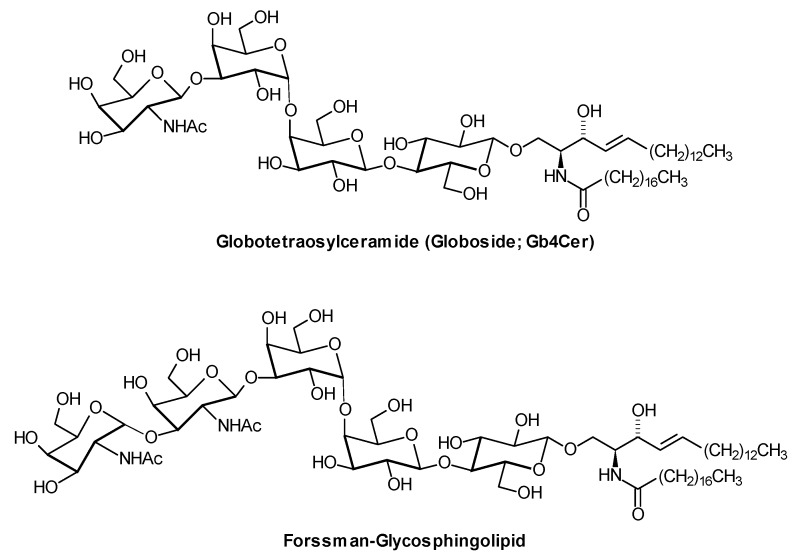
Structures of two globo-series GSLs, Gb4Cer and the Forssman-GSL [26]. Only one of the various lipoforms is shown. Together with ganglioside GM2, Gb4Cer is a major storage substance in Sandhoff disease (β-hexosaminidase β-subunit deficiency). In humans, the Forssman-GSL, a blood group antigen, is present only in trace amounts together with globo-series and Lewis-type blood group glycolipids in the kidney [49]. In sheep, it is mainly responsible for haemolysis of erythrocytes in the presence of Gb4Cer antiserum. For example, in MDCK (Madin Darby canine kidney) cells, it plays a role in maintaining apical membrane transport, is absent in the plasma membrane of unpolarized MDCK cells and becomes the major GSL in their fully polarized state [50].

In classical GSL analysis, mass spectrometry has been used for structural determination of GSLs after separation by TLC or HPLC [39]. While fast atom bombardment-MS has been frequently applied as ionization technique in the past [40], current methods rely largely on MALDI- and especially electrospray (ESI) ionization [41], in combination with additional methods like enzymatic degradation or staining by glycan-specific lectins [42]. Applications of MS to GSL analysis include ESI for analyzing the fragmentation of permethylated GSLs and of lithium ion-adducts of GSLs in the positive‐ion mode, collision‐induced mass spectrometry, and the analysis of gangliosides and sulfatides in the negative ion mode. Various methods have been developed for the analysis of neutral GSLs [43]. For example, an approach for the structural elucidation of neutral GSLs was based on low-energy collision-induced dissociation (CID) ESI/MS/MS of lithiated adducts [44]. MALDI-TOF MS with high-energy CID at 20 keV allowed the determination of the neutral GSLs GlcCer, GalCer, LacCer, Gb3Cer, and Forssman-GSL (Figure 7) in equine kidneys [45]. Methods for the structural identification of GSLs from sources such as tissues, body fluids, and cells are still under development, e.g., on the basis of multiple-stage MS fragmentation [46]. Their application to large sample numbers or to a more comprehensive determination of the glycosphingolipidome is far from being a routine practice. At present, method development, and their utilization to address analytical and clinical questions go in parallel. Isomeric GSL species are difficult to discriminate and quantify by MS; the separate quantification of GlcCer and GalCer species is achieved either within a shotgun approach (see below) using differences in the peak intensity ratio of the product ions at *m/z* = 179 and 89 [47], or after HPLC-separation by ESI-MS/MS [48].

### 2.1. Sample Preparation and Glycosphingolipid Extraction

GSLs can be isolated from tissues by chloroform-methanol extraction [51]. This step is even more critical than the extraction of other lipid classes: Early work revealed that recovery of GSLs is improved if small amounts of water are present in the extraction solvent [52]. Mild acidification has been applied in pre-analytics to dissociate gangliosides from hydrophobic peptides present in many samples, and also to reduce adduct formation with counter ions like sodium, potassium, ammonium, and others. For example, extraction with chloroform:methanol:water in a ratio of 5:5:1, taking into account an estimated presence of tissue water (70% for white brain matter, 80% for grey matter) has been used for the isolation of both, gangliosides and neutral GSLs [53]. A careful investigation of ganglioside recovery from erythrocytes revealed an uneven distribution of the analyte and led to the suggestion of 19 sample volumes of chloroform/methanol in the ratio of 1:2 in a one-step extraction [54]. If a partition step, such as that developed by Folch [55] with the system chloroform/methanol/aqueous solution, is applied, gangliosides partition into the aqueous phase. From there, their isolation by solid phase extraction, e.g., with an aminopropyl solid phase suitable also for separation of glycosylsphingosines [56], can be followed by separation into neutral and negatively charged species via anion exchange chromatography [57] such as DEAE-Sephadex [58], subsequent separation by HPLC or TLC, and quantification by mass spectrometry, or by staining and densitometry.

### 2.2. Glycosphingolipid Standards

Quantitative determination of analytes by MS requires the presence of calibrating substances, either as external standards, or chemically modified as internal standards. External standards need not be structurally different from the analytes and are therefore much more easily accessible. They can be used for external calibration or can be added in increasing concentrations to the analyte within the standard addition method [59]. In a recent example, eight different classes of gangliosides from the brains of rats were quantified using commercially available (native) external standards for GM1, GM2, GM3, GD3, GD1a, GD1b, GT1b, and GQ1b [60].

Internal standards contain rare alkyl chain combinations or the stable isotopes ^2^H or ^13^C, so that their *m/z*-values do not overlap with those of the analyte [61]. Physical and chemical properties of the standard should match those of the analyte as closely as possible. For the analysis of structurally complex lipids like GSLs including gangliosides, standards have to be chemically prepared in a laborious manner and are still not available for a number of analytes. For example, GSLs have been detected in a study of the lipidome of human plasma, but were not quantified because standard substances for more complex members were not available [62]. [2H4]- and [C_17_]ceramide trihexoside standards were prepared for Gb3Cer quantification [63]; [2H4]C_16_- and [2H47]C_24_-isoforms of GalCer, LacCer, Gb3Cer, sulfatide, sphingomyelin, gangliosides GM1, GM2, and GM3 have been synthesized as standard substances [64]. Standard substances for typically low abundance sulfatides have been prepared such as (18:1,14:0)sulfatide, (18:1,19:0)sulfatide, (18:1,27:0)sulfatide, 2-hydroxy fatty acid containing (18:1,h14:0)sulfatide, and the C_14_-, C_19_-, and C_27_- derivatives of sulfated LacCer, sulfated GA2, and C_19_-bis sulfated GA1 [65].

For the determination of the yeast lipidome, inositolphosphorylceramide, mannosyl-inositolphosphorylceramide, and mannosyl-diinositolphosphorylceramide standards were isolated from a double hydroxylase mutant yeast strain that synthesizes sphingolipids with dihydrosphingosine and a 26:0 fatty acid moiety [66]. Today, non-natural standard substances with either unusual chain lengths or stable isotopes are commercially available for GlcCer, GalCer, LacCer, Gb3Cer, GM3, GM2, GM1, sulfatide, and GalCer-3,6-disulfate.

### 2.3. Separation

Chromatographic separation techniques aside from MS analysis are especially valuable in lipidomics, when the combination of high pressure and small particle size allows high chromatographic resolution and / or enables separation of the analytes at reduced analysis times. GSLs are separated using different stationary phases, normal phase such as silica gel, which separates largely according to the head group, and reversed phase chromatography that separates mainly according to the hydrophobic part. A robust technique for separation of lipids that utilizes normal phase is TLC [67], a technology that has also been successfully combined with MALDI-MS [68]. TLC-separation in combination with immunodetection and an orthogonal mass spectrometric method such as IR-MALDI has turned out to be a very attractive technology [69]. Improvement of the existing technology for high throughput analyses will include miniaturization and automation of TLC separation and GSL identification with highly specific lectins, and the coupling to suitable MS techniques. This approach might become applicable for the very difficult determination of abundant polyglycosylceramides [70]. Neutral GSLs from mouse kidney, spleen, and small intestine have been analyzed by a combination of HPTLC and MALDI-MS [71]. For MS-analysis, separation by LC methods offers the advantage to reduce ionization suppression by other lipid species and allows for separation of isomeric and isobaric species that is difficult to achieve by MS methods with direct infusion alone. For example, separation and analysis of 182 neolacto series ganglioside species with sialic acids in α2,3 and α2,6 linkage from human granulocytes has been achieved by LC coupled online to nanoESI-MS [72]. Differences in the fragmentation patterns were applied previously for the analytical discrimination between these two types of gangliosides [73]. Also hydrophilic interaction liquid chromatography (HILIC [74]) has been used for GSL separation, such as in the determination of various lipids including HexCer and LacCer [75]. HILIC can be suitable when GSL analytes differ in their hydrophilic part. It can offer some advantages over RP-HPLC such as good peak shapes, short analysis times, and co-elution of analytes and their internal standards, but can suffer from poorer reproducibility of elution profiles. Also separation by capillary electrophoresis has been applied to GSL-analysis by ESI-MS [76].

### 2.4. Shotgun Lipidomics

The expression “shotgun lipidomics” describes the quantitative analysis of lipids by direct infusion of crude extracts in an ESI-MS source without a previous chromatographic separation [77]. This approach exploits the fact that manipulating ionization parameters in ESI-MS such as ionization mode or pH results in a preferential ionization of selected lipid groups or classes in the ionization source [78]. The “shotgun lipidomics” approach has been shown to be simple and effective for highly abundant lipids. However, less abundant lipid species can be overlooked due to ion suppression effects. To overcome this limitation, the crude extract needs to be processed prior to analysis. For example, it has been shown that a mild alkaline hydrolysis of a crude extract results in the elimination of the highly abundant glycerophophospholipids and consequently results in gaining access to the less abundant sphingolipid species [17]. Another possibility is to consider LC/MS methods [17].

### 2.5. Electrospray Ionization (ESI)

ESI is the most frequently applied ionization technique in GSL analysis by MS. During ESI, a spray of highly charged droplets is created from an analyte-containing solution by applying a high voltage at the end of a fine capillary needle [59,78,79]. Evaporation of the solvent is accompanied by emission of analyte ions into the gas phase, which are then transferred from the atmospheric-pressure segment to the high vacuum segment of the mass analyzer. Analytes are either infused directly into the interface via a syringe, or coupled to liquid chromatography (LC/ESI-MS). ESI can be conducted in a positive or a negative mode, as selected by the capillary voltage. Gangliosides and sulfatides are usually determined in the negative ion mode, sphingoid bases and ceramides can be identified by multiple reaction monitoring (see below) in the positive ion mode. ESI is an exceptionally mild ionization method that allows the formation of intact molecular ions with no or little fragmentation during ionization [59,78,79]. Fragmentation can occur in the ion-transport region of the ESI source (in-source fragmentation). At higher cone voltages, in source-fragmentation of sialic acids and sulfate is observed with gangliosides and sulfatides, respectively. The major limitations of ESI, which are adduct formation, restriction to polar solvents, and ion suppression when complex matrices are used, can be minimized by using nano-ESI-MS, a miniaturization of ESI-MS.

Coupling of RP-HPLC to ESI-MS allowed the separation of negatively charged GSLs such as sulfatides,  GM3, GM2,  GM1, GD3, GD1a, GD2, GD1b, GT1a (for structures, see Figure 5), GT1b, and  GQ1b in 25 min and their analysis at picomolar to femtomolar levels [80]. Sphingolipid profiling including determination of HexCer- and LacCer-species was achieved in human plasma and in lipoproteins after separation of lipid classes by HILIC [81,82,83]. Neutral GSLs from human erythrocytes were determined at the low femtomol level after nano-HPLC separation by ESI-QTOF MS. Modification of this fast and sensitive protocol also allowed the structural analysis and profiling of LacCer, Gb3Cer and globotetraosylceramide (globoside; Gb4Cer, for structure see Figure 7) species, including the determination of unsaturated and saturated ceramide moieties [84]. Very recently, Farwanah et al. presented a LC/ESI-MS method which allows the rapid separation and profiling of GlcCer, LacCer, Gb3Cer, and Gb4Cer [85].

### 2.6. Matrix-Assisted Laser Desorption/Ionization (MALDI)

MALDI is a mild ionization technique that has often been used for the qualitative analysis of larger molecules and biopolymers, and more recently also for the identification and the profiling of lipids. Unlike ESI, APCI, and APPI, MALDI ionizes the analyte of interest directly from a solid phase [59]. The analyte is co-crystallized together with an UV-active matrix on a metal target. Irradiation of suitable positions on the target with a pulsed laser leads to desorption and the ionization of the analyte. MALDI has the advantage of being less sensitive to salts. However, the nature of the used matrix and the heterogeneities of the created crystals can affect the results significantly, and the preparation of the matrix can be demanding. MALDI cannot be directly combined with liquid chromatography, but has been coupled to TLC. For the analysis of gangliosides by MALDI-MS, different matrices that lead to sodium- or potassium adducts of the analytes have been investigated [86], such as 6-azo-2-thiothymine/diammonium citrate [87]. MALDI coupled to nanoHPLC via an automatic spotting roboter allowed the analysis of complex GSL mixtures using 2,5-dihydroxybenzoic acid and 6-azo-2-thiothymine matrix systems [88]. Gasphase separation of lipid analytes on the basis of their ion mobility is an attractive tool for the high-throughput structural analysis in lipidomics [89]. The combination of MALDI and Ion mobility MS has been applied to the separation and analysis of HexCer (cerebrosides) [90] and gangliosides [91].

### 2.7. Other Ionization Techniques

Ionization techniques other than MALDI and ESI have been less often applied to the determination of GSLs. Atmospheric Pressure Chemical Ionization (APCI) is a form of chemical ionization, which is complementary to ESI in that it is usually used for the analysis of less polar lipids in LC eluates. In APCI, the analyte solution enters a sample capillary heated to high temperature in the range of 400 °C–500 °C. The combination of heat and a high flow rate of nitrogen leads to the formation of fine droplets of the analyte solution and a subsequent rapid evaporation of the solvent. A corona metal needle of high voltage at the end of the sample capillary transfers charges onto the analyte molecules [59,78,79]. An advantage of APCI is that less polar solvents such as hexane and chloroform do not significantly affect the ionization process, and adduct formation is less prominent in APCI than in ESI. A significant extent of in-source fragmentation limits its use especially for quantitative analysis. APCI has been occasionally applied to GSL analysis. For example, coupling of reverse phase chromatography to APCI enabled the determination of molecular weight, ceramide composition, and partial oligosaccharide sequence of 60 ng ganglioside [92]. Separation of cholesterol, sphingomyelin, and the neutral GSLs GlcCer, LacCer, Gb3Cer, and Gb4Cer in one run by normal phase LC was combined with densitometric quantification of these lipid classes and their profiling by APCI [85]. These GSLs are known to accumulate in inherited diseases of sphingolipid degradation, such as Gaucher-, Fabry-, and Sandhoff disease.

In Atmospheric Pressure Photo-Ionization (APPI), a modification of APCI, the corona needle is replaced by a UV lamp, which emits photons of 10 eV [59]. Since most lipid molecules have higher ionization energies than 10 eV, APPI is usually performed in a way in which the ionization segment is filled with a substance like acetone or toluene that has a lower ionization energy. Photons absorbed by the dopant molecules lead to the formation of radical cations, which transfer charge directly or via solvent-mediated ionization onto the analyte molecules [59]. Compared to APCI and ESI, APPI can have the advantage of lower detection limits and of higher signal intensities. APPI has been applied to the determination of neutral GSLs after separation by liquid chromatography on porous graphitic carbon [93].

### 2.8. Tandem Mass Spectrometry

**Figure 8:**
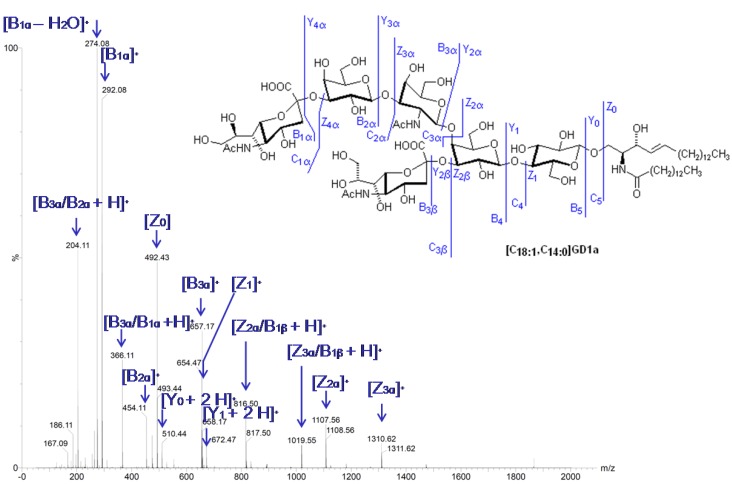
Collision induced dissociation (CID) of the ganglioside standard substance [C_18:1_,C_14:0_]GD1a. Fragmentation of the [M+2H]^2+^ signal at *m/z* = 891,37 in the positive mode is shown (M. Gantner, T. Kolter, unpublished). For the nomenclature of fragments, compare [97].

Tandem mass spectrometry (MS/MS) is the induced formation of fragments from the analyte of interest for structural elucidation or quantification. This is achieved by collision induced dissociation (CID), where *m/z*-selected molecular precursor ions collide with inert gas molecules such as helium or argon, which results in the dissociation of the analyte into fragments (Figure 8). In tandem-in-space MS, the molecular ions of the analyte are *m/z*-scanned, fragmented, and analyzed in different segments of the mass spectrometer. Such an approach can be performed in triple quadrupole mass analyzers, which consist of three quadrupoles (Q1, Q2, Q3) aligned in row, with Q2 as the collision cell in which fragmentation takes place. Here, different scan modes can be performed [59]. In the product ion scan mode, analyte species with a certain *m/z*-value are selected in Q1, enter the collision cell Q2, and the resulting fragments are scanned in Q3. Such a scan mode is usually performed when fragmentation conditions or/and characteristic fragments are searched for. If a characteristic fragment is already known, the precursor (or parent-) ions can be easily determined by a parent ion scan by focusing Q3 on these characteristic fragments and scanning for the corresponding precursor ions using Q1. Characteristic but not charged (neutral) and thus undetectable fragments are used in the so called neutral loss scan, where Q1 and Q3 are applied for scanning operations, but with a mass difference representing the neutral fragment lost during fragmentation. Multiple reaction monitoring (MRM) is a frequently used LC methodology for quantitative analysis. Here, Q1 is fixed to transmit precursor ions of a certain *m/z*-value, while Q3 is focused on a selected product ion. As a result, a certain transition is monitored. Q1 and Q3 can then be switched rapidly to other *m/z*-value pairs; a process which can be repeated multiple times. The advantages of MRM include good selectivity and sensitivity, as well as wide dynamic range.

In gangliosidoses, the ESI-MS/MS-detection of precursor ions of *m/z* = 290 that indicates the loss of *N*-acetylneuraminic acid has been used for the profiling of gangliosides [94]. A negatively charged precursor ion of *m/z* = 87 can be used at higher collision energy, together with other precursor ions such as the positively charged ions of HexNAc (*m/z* = 204), (HexNAc)_2_ (*m/z* = 407), or sphingosine (*m/z* = 264) [14]. Gangliosides GD3 and GM3 have been determined by monitoring a Neu5Ac-fragment of *m/z* = 290 in milk, after RP-separation via a C5-column and a standard addition technique [95]. [M−H]^−^- ions of sulfatides, the major storage substances in Metachromatic Leukodystrophy, were investigated by collisional-activated dissociation in a quadrupole ion-trap tandem mass spectrometer for assignments of sphingoid base and the fatty acid part of the ceramide anchor [96].

A disadvantage of triple quadrupole instruments is the low resolution power, which can be overcome by using hybrid mass spectrometers such as QTOFs (Quadrupole Time of Flight). Another drawback of triple quadrupole mass spectrometers is their inability to perform multiple stage fragmentations. Such fragmentations can be carried out in ion trap analyzers and in instruments with technical improvements derived therefrom. In principle, an ion trap analyzer consists of a ring electrode and two end cap electrodes with an oscillating trapping electrical field imposed on it [59]. Manipulation of the electrical field may lead to destabilization of the stable trajectories of trapped ions resulting in their ejection out of the mass analyzer. In ion traps, fragments are generated by colliding the analyte of interest with an inert gas in the ion trap analyzer itself. The resulting fragments can be fragmented n-times further (with n > 2). These multiple fragmentations are referred to as tandem in time mass spectrometry since the various fragments are generated in the same location but at different times. Another new development is the introduction of a fully automated thin chip microsprayer technology coupled to a high resolution hybrid quadrupole time-of-flight mass spectrometer for the mapping, sequencing, and structural elucidation of gangliosides. Using advanced chip-based setups, complex gangliosides such as GT1 were characterized after electrospray ionization in ion trap mass spectrometers [98]. In a high-throughput setup, a complete fragmentation analysis of gangliosides such as GT1b can be performed within three minutes [99].

### 2.9. Imaging Mass Spectrometry (IMS)

MS-based imaging techniques aim to visualize the distribution of molecules within tissue sections, including that of lipids [100]. To create spatial *m/z*- maps of the GSL analytes, MALDI-MS [101], and Secondary Ion MS (SIMS) [102] have been used, as well as Desorption electrospray ionization (DESI) [103]. In the case of the MALDI imaging, tissue sections embedded in suitable matrices are irradiated by nitrogen- or infrared laser beams. Heterogeneity of the matrix, extended acquisition times, chemical noise at low *m/z*, and restricted lateral resolution of 20–50 µm are shortcomings of the MALDI imaging approach. 

In SIMS, a surface is bombarded with a high energy ion beam, which leads to the sputtering of secondary ions that are collected and analyzed by MS [78]. Compared to MALDI, some advantages are no need for a matrix, a higher resolution in the sub-micrometer range, and less noise at low *m/z*-values. However, SIMS induces fragmentation of the analytes of interest, a restriction, which can limit its use. TOF-SIMS has been used to analyze the spatial distribution of sulfatide and other brain lipids [104], GalCer species in the white matter and the inner granular layer of rat cerebellum [105], and of GalCer- [106] and sulfatide-species [107] in rat cerebellum. Also DESI has been used to create two-dimensional images of the distribution of sphingolipid species in tissues [108].

MALDI imaging of ganglioside molecular species in the mouse hippocampus showed a differential distribution of C_18_- and C_20_-fatty acid-containing gangliosides [109]. Histological slices of brains from the mouse models of Tay-Sachs and Sandhoff disease were studied by MALDI imaging using an oscillating capillary nebulizer for matrix deposition on the surface of the sample [110]. Ganglioside profiles of coronal mouse brain sections have been determined with MALDI imaging using a 2,6-dihydroxyacetophenone/ammonium sulfate/heptafluorobutyric acid matrix to maximize the detection of all ganglioside species [111]. MALDI imaging using 9-aminoacridine as a matrix has been applied to kidneys from normal mice and mice with arylsulfatase A-deficiency, the animal model of Metachromatic Leukodystrophy. The study revealed a differential localization of sulfatides with identical ceramide anchors but different glycan-sulfate head groups, and of sulfatides with identical head groups, but different ceramide moieties [112]. In a nanoparticle-assisted way, distribution of sulfatide-species was determined in different layers of the dentate gyrus of rat hippocampus [113].

### 2.10. Indirect Methods

**Figure 9:**
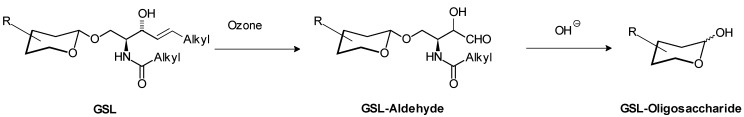
Oxidative cleavage of GSL and fragmentation of the resulting GSL-aldehydes. This gives access to GSL glycans.

Modern techniques of glycome profiling, for example after separation of isomeric glycan species by capillary electrophoresis, anion exchange chromatography, or other techniques [114], are not directly applicable to glycolipids, until after the chemical release of the sugar moiety. It has been known for a long time that the carbohydrate moiety of acetyl-protected GSLs can be liberated [26] by osmiumtetroxide/periodate-treatment [115] or of unprotected (native) GSLs by ozonolysis [116]. Both methods give rise to GSL aldehydes, which are subsequently fragmented by alkaline treatment (Figure 9). These GSL-aldehyde derivatives can also be isolated and immobilized, and for example transferred onto aminooxy-functionalized gold nanoparticles via chemical ligation in a glycoblotting technology, and directly profiled by MALDI-MS [117]. MALDI from gold nanoparticles can show higher ionization efficiency than from organic matrices. Also enzymatic methods can be applied for the preparation of GSL-derived oligosaccharides, such as a protocol using Rhodococcal endoglycoceramidase and leech ceramide glycanase [118]. Cell-derived GSL aldehydes have been linked to a heterobifunctional fluorescent tag, and coupled to glass slides to create shotgun microarrays. These were suitable for binding studies with toxins, antibodies, and sera from individuals with Lyme disease and mass spectrometry [119].

## 3. Identification of Interacting Molecules

Lipidomics has not only been understood as systems-level analysis and characterization of lipids, but includes also the determination of interacting molecules [120]. For the identification of interacting molecules, underivatized glycolipids can be separated by TLC, immobilized on hydrophobic surfaces, and probed with lectins, antibodies, pathogens, toxins, and whole cells. Such overlay techniques have been frequently applied to the characterization of GSLs as receptors for pathogens and toxins [121]. This type of GSL-profiling has also been combined with MS, such as MALDI for the structural characterization of pathogen receptors like those for Shiga toxins [122] or with IR-MALDI to determine neutral GSLs recognized by P-fimbriated *E. coli* bacteria [123]. TLC immuno-staining in combination with nano-ESI-MS and nano-ESI-MS/MS has been used to determine the structural profiles of Gb3Cer and Gb4Cer-binding Shiga toxin and antibodies [124]. In general, microwell adsorption, TLC overlay, and surface plasmon resonance spectroscopy are suitable techniques for the characterization of biopolymers interacting with the GSL glycan part [125]. Plasmon resonance for the identification of interacting moieties is also applicable after glycoblotting of the released GSL-glycans on gold particles [117].

## 4. Applications

### 4.1. Analysis of Lipidomes

MS techniques have been applied to the analysis of GSLs in the context of other questions. In some examples, the study of selected GSLs has been included in the lipidomic analysis of different systems. In a shotgun-based approach, the lipidome of Madin–Darby canine kidney cells was determined by Fourier transform MS analysis on a LTQ-Orbitrap instrument. The high mass accuracy of such instruments (of less than 2 ppm in non-hybride mode) enables highly confident identifications and structural assignments. More than 300 lipid species from 14 different lipid classes were measured from a single sample of 105 cells. Hexosylceramide, dihexosylceramide, and Forssman-GSL species were quantified in positive-ion-mode, and ganglioside GM3 species were quantified in the negative-ion-mode [126]. Because no synthetic standards were available for ganglioside GM3 and Forssman-GSL, the authors developed a quantification method that used standards purified from natural sources.

Changes in the lipidome of mouse macrophages in response to a highly specific ligand for Toll-like receptor 4 have been determined, including glucosylceramide and sulfatide [127]. Also the lipidomes of vesicular stomatitis virus, semliki forest virus, and the host plasma membrane have been studied by quantitative shotgun mass spectrometry [128]. 

### 4.2. Mutants and Other Functional Studies

Mutant cells and animals allow functional studies and have been applied to investigate the response of the systems to the lack of one or more lipid classes. Sulfatides of unusual structure have been characterized in the kidneys from mice with genetically engineered defects in ganglioside biosynthesis. A series of diagnostic fragments such as precursor ion of *m/z* = 97 for hydrogensulfate and others have been applied in a nanoESI MS/MS-approach [65]. A study of genetically engineered mice with defects in ganglioside biosynthesis revealed that polyunsaturated, fucosylated GSLs are essential for spermatogenesis and male mouse fertility [14]. Mass spectrometric profiles and quantitative amounts of HexCer, sulfatide, and other sphingolipids were determined in ceramide synthase 2 knockout mice [129]. LacCer content and fatty acid profile were determined in human neutrophils to study the role of LacCer molecular species for superoxide-generating and migrating abilities [130].

### 4.3. Inherited Diseases

GSL storage diseases [131] are due to genetic defects in enzymes and other proteins required for lysosomal GSL degradation. Determination of GSL levels is of special interest for diagnosis and monitoring therapy [132] of these diseases, where GSL substrates of the deficient enzymes accumulate in different organs. For example, a GSL of the globo-series, Gb3Cer, is the major storage substance in Fabry disease. It accumulates when the enzyme required for the cleavage of the terminal galactosyl residue, α-galactosidase A, is deficient. With the aid of synthetic internal standards, plasma levels of Gb3Cer were determined in patients with Fabry disease. Gb3Cer levels have also been determined by ESI using stearoyl-d_35_-Gb3Cer as internal standard. Isoform profiles of Gb3Cer were recorded in urine [133] and plasma [134] of Fabry patients, heterozygote carriers, and probands. LC-MS has also been applied to monitor treatment of Fabry patients with enzyme replacement therapy [135]. MS-determination of Gb3Cer in urine was demonstrated as a screening method that allows detection of female hemizygotes [136]. During the determination of Gb3Cer-levels in Fabry patients and healthy individuals using C_17_-Gb3Cer as internal standard, a study using different mass spectrometers revealed that careful adjustments are required to obtain identical isoform intensities and quantitative results [137].

In ESI, sulfatides can be detected by a *m/z* = 97 in the precursor ion scan, which has been applied to determine urinary sulfatides in Metachromatic Leukodystrophy [138].

HexCer from vitreous bodies from a patient with Gaucher disease was analyzed by delayed extraction MALDI [139], as well as a quantitative determination of HexCer and sphingomyelin from liver and spleen specimens derived from patients with Niemann-Pick and Gaucher disease [140]. The levels of HexCer and other lipids were determined in Gaucher fibroblasts using the MRM approach. The analysis revealed in part a significant secondary elevation of ceramide, dihexosylceramide, trihexosylceramide, sphingomyelin, phosphatidylcholine, phosphatidylglycerol, and phosphatidylinositol, especially in type 2 Gaucher disease. ESI-MS determination after sub-cellular fractionation showed that the accumulation was not restricted to the lysosomal compartment [141].

**Figure 10:**
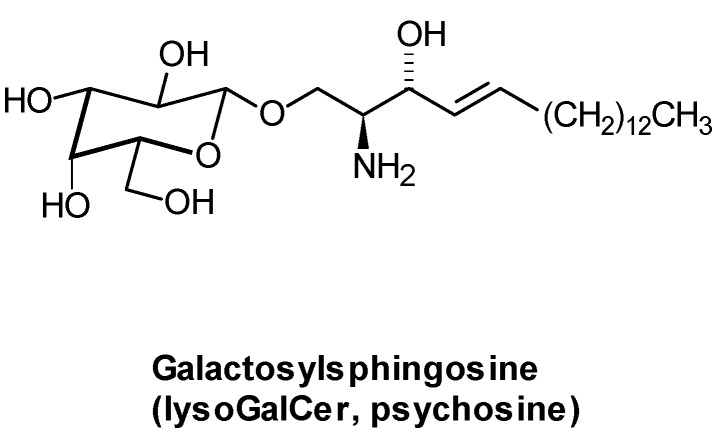
Structure of Galactosylsphingosine, a toxic substance that accounts for the pathogenesis of Krabbe disease (GalCer-β-galactosidase deficiency, Globoid cell leukodystrophy).

Lysoglycosphingolipids are toxic site products of GSL biosynthesis and are elevated in patients with sphingolipidoses [142]. Especially the pathogenesis of Krabbe disease is governed by the accumulation of galactosylsphingosine (Psychosine, Figure 10) in oligodendrocytes. In Fabry disease, globotriaosylsphingosine levels in urine [143], tissue and plasma [144] have been determined as biomarkers for the disease and monitoring of enzyme replacement therapy. More recently, lysoGSLs have also been detected and determined in healthy probands and those suffering from acquired diseases (see below). In patients with coronary heart disease, they occur in HDL [145], and seem to have beneficial effects on the heart. 

### 4.4. Acquired Diseases

Glycosphingolipid levels are altered not only in sphingolipidoses, but in a variety of other diseases [146]. Determination of GSL molecular species and their correlation with acquired disease states are rare, but some attempts have been made to correlate GSL molecular species with physiological and pathophysiological conditions. For example, determination of GlcCer and LacCer in different organs revealed a correlation between aging and certain molecular GSL species, such as C_14_- and C_16_-LacCer. This study was carried out in rodents and in cells from human probands. Levels were elevated as much as 8 and 12-fold in mice and in fibroblasts from elderly human individuals; an effect that could be reversed by caloric restriction [147].

#### 4.4.1. Folding Diseases

GSL-levels have been correlated with folding diseases, such as Alzheimer and prion diseases. The presence of gangliosides appears to be required for the formation of amyloid fibrils, a hallmark of Alzheimer diseases [148], and antibodies against ganglioside GM1 prevent amyloid fibril formation [149]. Shotgun Lipidomics revealed changes in the lipid composition of the brain of Alzheimer disease patients, especially of sulfatides, ceramides, and plasmalogens [150].

#### 4.4.2. Diabetes

Several lines of evidence support the view that ganglioside GM3 downregulates the insulin receptor. Although an interaction between lysine-944 of the insulin receptor and the carboxylic acid moiety of ganglioside GM3 seems to play a role, the molecular details of this phenomenon are not clear [151]. Accordingly, elevated GM3 levels have been observed in the serum of patients with hyperglycemia, hyperlipidemia, type 2 diabetes, and obesity [152]. Determination of serum GM3 levels might be suitable as a diagnostic marker for such metabolic diseases. In addition, pharmacological inhibition of ganglioside biosynthesis has been suggested to be a new approach for the treatment of diabetes [153], although–at least in liver–questions about the molecular bases of the observed effects remain [154]. In animal models of type 1 diabetes, hexosylceramide species containing nervonic acid (24:1) were reduced in circulation, liver, and heart, and also in response to a high fat diet [155].

#### 4.4.3. Cancer

The different steps of sphingolipid and GSL metabolism are altered in cancer [156]. GSLs also influence the regulation of genes that affect cancer in terms of tumorigenesis, metastasis, or tumor response to treatment [157]. For example, there is evidence for a tumor-associated occurrence of ganglioside Hanganutziu-Deicher antigens. Based on this, vaccines against *N*-glycolylneuraminic acid-containing ganglioside GM3 have been tested in clinical trials for cancer-treatment [158].

**Figure 11:**
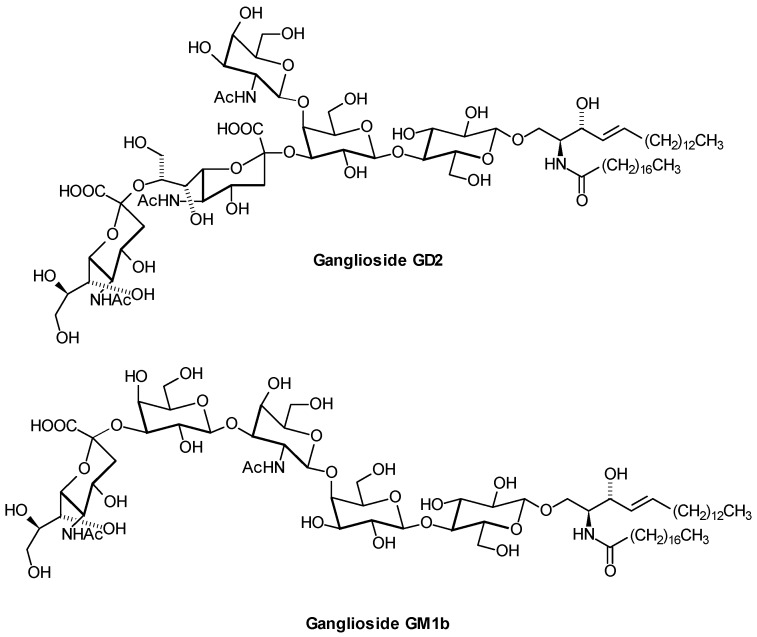
Structures of gangliosides GD2 and GM1b. Only one of the various lipoforms is shown.

Thirty-one different ganglioside species from murine lymphoma cells have been separated from classical gangliosides by nanoHPLC coupled online to ESI-QTOF MS. Among them were 18 Neu5Gc-containing gangliosides and 13 Neu5Ac-containing species of ganglioside GM1b (Figure 11) and GalNAc-GM1b [22]. Aberrant sialylation has been associated with malignancy: Expression of the four types of human sialidases have different impact on cancer malignancy [159]. Ganglioside GD2 (Figure 11) is regarded as a tumor-associated antigen present on human neuroblastoma cells, and clinical trials have been conducted using anti-GD2 antibodies [160]. Neuropathic pain reactions in the immunotherapy were one of the major side-effects [161]. In pancreatic and also in colon cancer, MS analysis revealed increased levels of Gb3Cer with long chain (C_24_), short chain fatty acids (C_16_), and hydroxylated fatty acid lipoforms in malignant tissues [162]. In cancer, Gb3Cer is elevated on the surface of tumor cells, where it facilitates the anchorage of heat shock-protein 70 in the plasma membrane [163]. Modified gangliosides like fucosylated GM3 and GM4, and acetylated GM1 and GM3 were determined in brain tumors using chip-based nanoelectrospray MS [164].

Combined bioinformatic and lipidomic approaches make use of the data on gene expression in cancer and other diseases to predict differences in sphingolipid and GSL composition. This has been applied to breast cancer cell lines [165] and to ovarian cancer, where a combination of MALDI imaging and transcriptomic data indicated that sulfatides are elevated [166].

## 5. Outlook

Lipidomics aims to determine the lipid profile of a given system and to integrate this information with genomic, transcriptomic, and proteomic data. Despite considerable progress in method development and instrumentation, this is currently not achieved for GSLs even by the leading laboratories. Due to the structural complexity of the glycosphingolipidome, even a comprehensive GSL analysis is still a tremendous challenge and not possible within routine analytical work. Promising developments as summarized in this review include studies that correlate profiles of GSL subsets with human diseases, that address the spatial distribution of analytes, as well as the extension of analytical work towards more rare and potentially regulatory GSL species.

## References

[1] Chester M.A. (1997). Nomenclature of glycolipids. Pure Appl. Chem..

[2] Merrill A.H. (2011). Sphingolipid and glycosphingolipid metabolic pathways in the era of sphingolipidomics. Chem. Rev..

[3] Fahy E., Subramaniam S., Brown H.A., Glass C.K., Merrill A.H., Murphy R.C., Raetz C.R., Russell D.W., Seyama Y., Shaw W. (2005). A comprehensive classification system for lipids. J. Lipid Res..

[4] Wenk M.R. (2010). Lipidomics: New tools and applications. Cell.

[5] Jung H.R., Sylvanne T., Koistinen K.M., Tarasov K., Kauhanen D., Ekroos K. (2011). High throughput quantitative molecular lipidomics. Biochim. Biophys. Acta.

[6] Hart G.W., Copeland R.J. (2010). Glycomics hits the big time. Cell.

[7] Rakus J.F., Mahal L.K.  (2011). New technologies for glycomic analysis: Toward a systematic understanding of the glycome. Annu. Rev. Anal. Chem. (Palo Alto Calif).

[8] Gupta G., Surolia A., Sampathkumar S.G. (2010). Lectin microarrays for glycomic analysis. OMICS.

[9] Rillahan C.D., Paulson J.C. (2011). Glycan microarrays for decoding the glycome. Annu. Rev. Biochem..

[10] Zaia J. (2010). Mass spectrometry and glycomics. OMICS.

[11] Kolter T., Proia R.L., Sandhoff K. (2002). Combinatorial ganglioside biosynthesis. J. Biol. Chem..

[12] Levery S.B. (2005). Glycosphingolipid structural analysis and glycosphingolipidomics. Mass Spectrom. Modified Proteins Glycoconjugates.

[13] Teufel A., Maass T., Galle P.R., Malik N. (2009). The longevity assurance homologue of yeast lag1 (Lass) gene family (review). Int. J. Mol. Med..

[14] Sandhoff R., Geyer R., Jennemann R., Paret C., Kiss E., Yamashita T., Gorgas K., Sijmonsma T.P., Iwamori M., Finaz C. (2005). Novel class of glycosphingolipids involved in male fertility. J. Biol. Chem..

[15] Schauer R. (2009). Sialic acids as regulators of molecular and cellular interactions. Curr. Opin. Struct. Biol..

[16] Riboni L., Sonnino S., Acquotti D., Malesci A., Ghidoni R., Egge H., Mingrino S., Tettamanti G. (1986). Natural occurrence of ganglioside lactones. Isolation and characterization of GD1b inner ester from adult human brain. J. Biol. Chem..

[17] Jiang X., Cheng H., Yang K., Gross R.W., Han X. (2007). Alkaline methanolysis of lipid extracts extends shotgun lipidomics analyses to the low-abundance regime of cellular sphingolipids. Anal. Biochem..

[18] He H., Conrad C.A., Nilsson C.L., Ji Y., Schaub T.M., Marshall A.G., Emmett M.R. (2007). Method for lipidomic analysis: p53 expression modulation of sulfatide, ganglioside, and phospholipid composition of U87 MG glioblastoma cell. Anal. Chem..

[19] Vukelic Z., Kalanj-Bognar S., Froesch M., Bindila L., Radic B., Allen M., Peter-Katalinic J., Zamfir A.D. (2007). Human gliosarcoma-associated ganglioside composition is complex and distinctive as evidenced by high-performance mass spectrometric determination and structural characterization. Glycobiology.

[20] Kohla G., Stockfleth E., Schauer R. (2002). Gangliosides with *O*-acetylated sialic acids in tumors of neuroectodermal origin. Neurochem. Res..

[21] Herrler G., Schwegmann-Wessels C. (2006). Sialic acids as receptor determinants for coronaviruses. Glycoconjugate J..

[22] Zarei M., Muthing J., Peter-Katalinic J., Bindila L. (2010). Separation and identification of GM1b pathway Neu5Ac- and Neu5Gc gangliosides by on-line nanoHPLC-QToF MS and tandem MS: Toward glycolipidomics screening of animal cell lines. Glycobiology.

[23] Svennerholm L. (1963). Chromatographic separation of human brain gangliosides. J. Neurochem..

[24] Posse de Chaves E., Sipione S. (2010). Sphingolipids and gangliosides of the nervous system in membrane function and dysfunction. FEBS Lett..

[25] Yu R.K., Nakatani Y., Yanagisawa M. (2009). The role of glycosphingolipid metabolism in the developing brain. J. Lipid Res..

[26] Hakomori S.I. (1780). Structure and function of glycosphingolipids and sphingolipids: Recollections and future trends. Biochim. Biophys. Acta.

[27] Schnaar R.L., Mehta N.R., Nguyen T., Griffin J.W. (2010). Ganglioside engagement by myelin-associated glycoprotein (MAG) protects axons from acute toxic insults. J. Neurochem..

[28] Schengrund C.L. (2010). Lipid rafts: Keys to neurodegeneration. Brain Res. Bull..

[29] Yamashita T., Hashiramoto A., Haluzik M., Mizukami H., Beck S., Norton A., Kono M., Tsuji S., Daniotti J.L., Werth N. (2003). Enhanced insulin sensitivity in mice lacking ganglioside GM3. Proc. Natl. Acad. Sci. USA.

[30] Eggeling C., Ringemann C., Medda R., Schwarzmann G., Sandhoff K., Polyakova S., Belov V.N., Hein B., von Middendorff C., Schonle A. (2009). Direct observation of the nanoscale dynamics of membrane lipids in a living cell. Nature.

[31] Sandhoff K., Gallala H.D. (2008). Principles of microdomain formation in biological membranes-Are there lipid liquid ordered domains in living cellular membranes?. Trends Glycosci. Glycotechnol..

[32] Malorni W., Garofaloa T., Tinari A., Matarrese P., Giammarioli A.M., Manganelli V., Ciarlo L., Misasi R., Sorice M. (2007). Do mitochondria act as "Cargo boats" in the journey of GD3 to the nucleus during apoptosis?. FEBS Lett..

[33] Hanada K. (2005). Sphingolipids in infectious diseases. Jpn. J. Infect. Dis..

[34] Holgersson J., Stromberg N., Breimer M.E. (1988). Glycolipids of human large intestine: Difference in glycolipid expression related to anatomical localization, epithelial/non-epithelial tissue and the ABO, Le and Se phenotypes of the donors. Biochimie.

[35] Zumbrun S.D., Hanson L., Sinclair J.F., Freedy J., Melton-Celsa A.R., Rodriguez-Canales J., Hanson J.C., O'Brien A.D. (2010). Human intestinal tissue and cultured colonic cells contain globotriaosylceramide synthase mRNA and the alternate Shiga toxin receptor globotetraosylceramide. Infect. Immun..

[36] Schweppe C.H., Hoffmann P., Nofer J.R., Pohlentz G., Mormann M., Karch H., Friedrich A.W., Muthing J. (2010). Neutral glycosphingolipids in human blood: A precise mass spectrometry analysis with special reference to lipoprotein-associated Shiga toxin receptors. J. Lipid Res..

[37] Senn H.J., Orth M., Fitzke E., Wieland H., Gerok W.  (1989). Gangliosides in normal human serum. Concentration, pattern and transport by lipoproteins. Eur. J. Biochem..

[38] Kusunoki S., Kaida K. (2011). Antibodies against ganglioside complexes in Guillain-Barre syndrome and related disorders. J. Neurochem..

[39] Haynes C.A., Allegood J.C., Park H., Sullards M.C. (2009). Sphingolipidomics: Methods for the comprehensive analysis of sphingolipids. J. Chromatogr. B Analyt. Technol. Biomed. Life Sci..

[40] Egge H., Peter-Katalinic J., Reuter G., Schauer R., Ghidoni R., Sonnino S., Tettamanti G. (1985). Analysis of gangliosides using fast atom bombardment mass spectrometry. Chem. Phys. Lipids.

[41] Chen Y.F., Liu Y., Sullards M.C., Merrill A.H. (2010). An Introduction to Sphingolipid Metabolism and Analysis by New Technologies. Neuromol. Med..

[42] Muthing J., Distler U. (2010). Advances on the compositional analysis of glycosphingolipids combining thin-layer chromatography with mass spectrometry. Mass Spectrom Rev..

[43] Farwanah H., Kolter T., Sandhoff K. (1811). Mass spectrometric analysis of neutral sphingolipids: Methods, applications, and limitation. Biochim. Biophys. Acta.

[44] Hsu F.F., Turk J. (2001). Structural determination of glycosphingolipids as lithiated adducts by electrospray ionization mass spectrometry using low-energy collisional-activated dissociation on a triple stage quadrupole instrument. J. Am. Soc. Mass Spectrom..

[45] Tanaka K., Yamada M., Tamiya-Koizumi K., Kannagi R., Aoyama T., Hara A., Kyogashima M. (2011). Systematic analyses of free ceramide species and ceramide species comprising neutral glycosphingolipids by MALDI-TOF MS with high-energy CID. Glycoconjugate J..

[46] Zamfir A.D., Vukelic Z., Schneider A., Sisu E., Dinca N., Ingendoh A. (2007). A novel approach for ganglioside structural analysis based on electrospray multiple-stage mass spectrometry. J. Biomol. Tech..

[47] Han X., Cheng H. (2005). Characterization and direct quantitation of cerebroside molecular species from lipid extracts by shotgun lipidomics. J. Lipid Res..

[48] Merrill A.H., Sullards M.C., Allegood J.C., Kelly S., Wang E. (2005). Sphingolipidomics: High-throughput, structure-specific, and quantitative analysis of sphingolipids by liquid chromatography tandem mass spectrometry. Methods.

[49] Holgersson J., Jovall P.A., Samuelsson B.E., Breimer M.E.  (1991). Blood group type glycosphingolipids of human kidneys. Structural characterization of extended globo-series compounds. Glycoconjugate J..

[50] Simons K., Sampaio J.L. (2011). Membrane organization and lipid rafts. Cold Spring Harb. Perspect. Biol..

[51] Lacomba R., Salcedo J., Alegria A., Jesus Lagarda M., Barbera R., Matencio E. (2010). Determination of sialic acid and gangliosides in biological samples and dairy products: A review. J. Pharm. Biomed. Anal..

[52] Svennerholm L., Fredman P. (1980). A procedure for the quantitative isolation of brain gangliosides. Biochim. Biophys. Acta.

[53] Byrne M.C., Sbaschnig-Agler M., Aquino D.A., Sclafani J.R., Ledeen R.W. (1985). Procedure for isolation of gangliosides in high yield and purity: Simultaneous isolation of neutral glycosphingolipids. Anal. Biochem..

[54] Wang W.Q., Gustafson A. (1995). Ganglioside extraction from erythrocytes: A comparison study. Acta Chem. Scand..

[55] Folch J., Lees M., Sloane Stanley G.H. (1957). A simple method for the isolation and purification of total lipids from animal tissues. J. Biol. Chem..

[56] Bodennec J., Pelled D., Futerman A.H. (2003). Aminopropyl solid phase extraction and 2 D TLC of neutral glycosphingolipids and neutral lysoglycosphingolipids. J. Lipid Res..

[57] Manzi A.E., Hayes B.K.  (2001). HPLC methods for the fractionation and analysis of negatively charged oligosaccharides and gangliosides. Curr. Protoc. Mol. Biol..

[58] Yu R.K., Ledeen R.W. (1972). Gangliosides of human, bovine, and rabbit plasma. J. Lipid Res..

[59] Dass C. (2007). Fundamentals of Contemporary Mass Spectrometry.

[60] Fong B., Norris C., Lowe E., McJarrow P. (2009). Liquid chromatography-high-resolution mass spectrometry for quantitative analysis of gangliosides. Lipids.

[61] Moore J.D., Caufield W.V., Shaw W.A. (2007). Quantitation and standardization of lipid internal standards for mass spectroscopy. Meth. Enzymology.

[62] Quehenberger O., Armando A.M., Brown A.H., Milne S.B., Myers D.S., Merrill A.H., Bandyopadhyay S., Jones K.N., Kelly S., Shaner R.L. (2010). Lipidomics reveals a remarkable diversity of lipids in human plasma. J. Lipid Res..

[63] Mills K., Johnson A., Winchester B. (2002). Synthesis of novel internal standards for the quantitative determination of plasma ceramide trihexoside in Fabry disease by tandem mass spectrometry. FEBS Lett..

[64] Mills K., Eaton S., Ledger V., Young E., Winchester B. (2005). The synthesis of internal standards for the quantitative determination of sphingolipids by tandem mass spectrometry. Rapid Commun. Mass Spectrom..

[65] Sandhoff R., Hepbildikler S.T., Jennemann R., Geyer R., Gieselmann V., Proia R.L., Wiegandt H., Grone H.J. (2002). Kidney sulfatides in mouse models of inherited glycosphingolipid disorders: Determination by nano-electrospray ionization tandem mass spectrometry. J. Biol. Chem..

[66] Ejsing C.S., Sampaio J.L., Surendranath V., Duchoslav E., Ekroos K., Klemm R.W., Simons K., Shevchenko A. (2009). Global analysis of the yeast lipidome by quantitative shotgun mass spectrometry. Proc. Natl. Acad. Sci. USA.

[67] Fuchs B., Suss R., Teuber K., Eibisch M., Schiller J. (1218). Lipid analysis by thin-layer chromatography--a review of the current state. J. Chromatogr. A.

[68] Fuchs B., Suss R., Schiller J. (2010). An update of MALDI-TOF mass spectrometry in lipid research. Prog. Lipid Res..

[69] Rohlfing A., Muthing J., Pohlentz G., Distler U., Peter-Katalinic J., Berkenkamp S., Dreisewerd K. (2007). IR-MALDI-MS analysis of HPTLC-separated phospholipid mixtures directly from the TLC plate. Anal. Chem..

[70] Meisen I., Mormann M., Muthing J. (2011). Thin-layer chromatography, overlay technique and mass spectrometry: A versatile triad advancing glycosphingolipidomics. Biochim. Biophys. Acta.

[71] Suzuki A., Miyazaki M., Matsuda J., Yoneshige A. (2011). High-performance thin-layer chromatography/mass spectrometry for the analysis of neutral glycosphingolipids. Biochim. Biophys. Acta.

[72] Kirsch S., Muthing J., Peter-Katalinic J., Bindila L. (2009). On-line nano-HPLC/ESI QTOF MS monitoring of alpha2-3 and alpha2-6 sialylation in granulocyte glycosphingolipidome. Biol. Chem..

[73] Meisen I., Peter-Katalinic J., Muthing J. (2003). Discrimination of neolacto-series gangliosides with alpha2-3- and alpha2-6-linked *N*-acetylneuraminic acid by nanoelectrospray ionization low-energy collision-induced dissociation tandem quadrupole TOF MS. Anal. Chem..

[74] Alpert A.J. (1990). Hydrophilic-interaction chromatography for the separation of peptides, nucleic acids and other polar compounds. J. Chromatogr..

[75] Scherer M., Leuthauser-Jaschinski K., Ecker J., Schmitz G., Liebisch G. (2010). A rapid and quantitative LC-MS/MS method to profile sphingolipids. J. Lipid Res..

[76] Zamfir A., Vukelic Z., Peter-Katalinic J. (2002). A capillary electrophoresis and off-line capillary electrophoresis/electrospray ionization-quadrupole time of flight-tandem mass spectrometry approach for ganglioside analysis. Electrophoresis.

[77] Han X., Gross R.W. (2005). Shotgun lipidomics: Electrospray ionization mass spectrometric analysis and quantitation of cellular lipidomes directly from crude extracts of biological samples. Mass Spectrom. Rev..

[78] Bou Khalil M., Hou W., Zhou H., Elisma F., Swayne L.A., Blanchard A.P., Yao Z., Bennett S.A., Figeys D. (2010). Lipidomics era: Accomplishments and challenges. Mass Spectrom. Rev..

[79] Farwanah H., Kolter T. (2007). Lipidomics. Wiley Encyclopedia of Chemical Biology.

[80] Ikeda K., Shimizu T., Taguchi R. (2008). Targeted analysis of ganglioside and sulfatide molecular species by LC/ESI-MS/MS with theoretically expanded multiple reaction monitoring. J. Lipid Res..

[81] Hammad S.M., Pierce J.S., Soodavar F., Smith K.J., Al Gadban M.M., Rembiesa B., Klein R.L., Hannun Y.A., Bielawski J., Bielawska A. (2010). Blood sphingolipidomics in healthy humans: Impact of sample collection methodology. J. Lipid Res..

[82] Liebisch G., Scherer M., Bottcher A., Schmitz G.  (2011). Sphingolipid profiling of human plasma and FPLC-separated lipoprotein fractions by hydrophilic interaction chromatography tandem mass spectrometry. Biochimica Et Biophysica Acta-Mol. Cell Biol. Lipids.

[83] Scherer M., Bottcher A., Liebisch G. (2011). Lipid profiling of lipoproteins by electrospray ionization tandem mass spectrometry. Biochim. Biophys. Acta.

[84] Kirsch S., Zarei M., Cindric M., Muthing J., Bindila L., Peter-Katalinic J. (2008). On-line nano-HPLC/ESI QTOF MS and tandem MS for separation, detection, and structural elucidation of human erythrocytes neutral glycosphingolipid mixture. Anal. Chem..

[85] Farwanah H., Wirtz J., Kolter T., Raith K., Neubert R.H., Sandhoff K. (2009). Normal phase liquid chromatography coupled to quadrupole time of flight atmospheric pressure chemical ionization mass spectrometry for separation, detection and mass spectrometric profiling of neutral sphingolipids and cholesterol. J. Chromatogr. B Analyt. Technol. Biomed. life Sci..

[86] Ivleva V.B., Elkin Y.N., Budnik B.A., Moyer S.C., O'Connor P.B., Costello C.E. (2004). Coupling thin-layer chromatography with vibrational cooling matrix-assisted laser desorption/ionization Fourier transform mass spectrometry for the analysis of ganglioside mixtures. Anal. Chem..

[87] Zarei M., Bindila L., Souady J., Dreisewerd K., Berkenkamp S., Muthing J., Peter-Katalinic J. (2008). A sialylation study of mouse brain gangliosides by MALDI a-TOF and o-TOF mass spectrometry. J. Mass Spectrom..

[88] Zarei M., Kirsch S., Muthing J., Bindila L., Peter-Katalinic J. (2008). Automated normal phase nano high performance liquid chromatography/matrix assisted laser desorption/ionization mass spectrometry for analysis of neutral and acidic glycosphingolipids. Anal. Bioanal. Chem..

[89] Kliman M., May J.C., McLean J.A. (2011). Lipid analysis and lipidomics by structurally selective ion mobility-mass spectrometry. Biochim. Biophys. Acta.

[90] Jackson S.N., Ugarov M., Egan T., Post J.D., Langlais D., Albert Schultz J., Woods A.S. (2007). MALDI-ion mobility-TOFMS imaging of lipids in rat brain tissue. J. Mass Spectrom..

[91] Jackson S.N., Colsch B., Egan T., Lewis E.K., Schultz J.A., Woods A.S. (2011). Gangliosides' analysis by MALDI-ion mobility MS. Analyst.

[92] Kushi Y., Rokukawa C., Numajir Y., Kato Y., Handa S. (1989). Analysis of underivatized glycosphingolipids by high-performance liquid chromatography/atmospheric pressure ionization mass spectrometry. Anal. Biochem..

[93] Roy S., Delobel A., Gaudin K., Touboul D., Germain D.P., Baillet A., Prognon P., Laprevote O., Chaminade P. (2006). Liquid chromatography on porous graphitic carbon with atmospheric pressure photoionization mass spectrometry and tandem mass spectrometry for the analysis of glycosphingolipids. J. Chromatogr. A.

[94] Tsui Z.C., Chen Q.R., Thomas M.J., Samuel M., Cui Z. (2005). A method for profiling gangliosides in animal tissues using electrospray ionization-tandem mass spectrometry. Anal. Biochem..

[95] Sorensen L.K. (2006). A liquid chromatography/tandem mass spectrometric approach for the determination of gangliosides GD3 and GM3 in bovine milk and infant formulae. Rapid Commun. Mass Spectrom..

[96] Hsu F.F., Turk J. (2004). Studies on sulfatides by quadrupole ion-trap mass spectrometry with electrospray ionization: Structural characterization and the fragmentation processes that include an unusual internal galactose residue loss and the classical charge-remote fragmentation. J. Am. Soc. Mass Spectrom..

[97] Adams J., Ann Q.H. (1993). Structure Determination of Sphingolipids by Mass-Spectrometry. Mass Spectrom. Rev..

[98] Vukelic Z., Zamfir A.D., Bindila L., Froesch M., Peter-Katalinic J., Usuki S., Yu R.K. (2005). Screening and sequencing of complex sialylated and sulfated glycosphingolipid mixtures by negative ion electrospray Fourier transform ion cyclotron resonance mass spectrometry. J. Am. Soc. Mass Spectrom..

[99] Serb A., Schiopu C., Flangea C., Sisu E., Zamfir A.D. (2009). Top-down glycolipidomics: Fragmentation analysis of ganglioside oligosaccharide core and ceramide moiety by chip-nanoelectrospray collision-induced dissociation MS2-MS6. J. Mass Spectrom..

[100] Goto-Inoue N., Hayasaka T., Zaima N., Setou M. (2011). Imaging mass spectrometry for lipidomics. Biochim. Biophys. Acta.

[101] Fernandez J.A., Ochoa B., Fresnedo O., Giralt M.T., Rodriguez-Puertas R. (2011). Matrix-assisted laser desorption ionization imaging mass spectrometry in lipidomics. Anal. Bioanal. Chem..

[102] Passarelli M.K., Winograd N. (2011). Lipid imaging with time-of-flight secondary ion mass spectrometry (ToF-SIMS). Biochim. Biophys. Acta.

[103] Eberlin L.S., Ferreira C.R., Dill A.L., Ifa D.R., Cooks R.G. (2011). Desorption electrospray ionization mass spectrometry for lipid characterization and biological tissue imaging. Biochim. Biophys. Acta.

[104] Sjovall P., Lausmaa J., Johansson B. (2004). Mass spectrometric imaging of lipids in brain tissue. Anal. Chem..

[105] Borner K., Nygren H., Hagenhoff B., Malmberg P., Tallarek E., Mansson J.E. (1761). Distribution of cholesterol and galactosylceramide in rat cerebellar white matter. Biochim. Biophys. Acta.

[106] Nygren H., Borner K., Hagenhoff B., Malmberg P., Mansson J.E. (2005). Localization of cholesterol, phosphocholine and galactosylceramide in rat cerebellar cortex with imaging TOF-SIMS equipped with a bismuth cluster ion source. Biochim. Biophys. Acta.

[107] Pernber Z., Richter K., Mansson J.E., Nygren H. (2007). Sulfatide with different fatty acids has unique distributions in cerebellum as imaged by time-of-flight secondary ion mass spectrometry (TOF-SIMS). Biochim. Biophys. Acta.

[108] Manicke N.E., Nefliu M., Wu C., Woods J.W., Reiser V., Hendrickson R.C., Cooks R.G. (2009). Imaging of lipids in atheroma by desorption electrospray ionization mass spectrometry. Anal. Chem..

[109] Sugiura Y., Shimma S., Konishi Y., Yamada M.K., Setou M. (2008). Imaging mass spectrometry technology and application on ganglioside study; visualization of age-dependent accumulation of C20-ganglioside molecular species in the mouse hippocampus. PLoS One.

[110] Chen Y., Allegood J., Liu Y., Wang E., Cachon-Gonzalez B., Cox T.M., Merrill A.H., Sullards M.C. (2008). Imaging MALDI mass spectrometry using an oscillating capillary nebulizer matrix coating system and its application to analysis of lipids in brain from a mouse model of Tay-Sachs/Sandhoff disease. Anal. Chem..

[111] Colsch B., Woods A.S. (2010). Localization and imaging of sialylated glycosphingolipids in brain tissue sections by MALDI mass spectrometry. Glycobiology.

[112] Marsching C., Eckhardt M., Grone H.J., Sandhoff R., Hopf C. (2011). Imaging of complex sulfatides SM3 and SB1a in mouse kidney using MALDI-TOF/TOF mass spectrometry. Anal. Bioanal. Chem..

[113] Ageta H., Asai S., Sugiura Y., Goto-Inoue N., Zaima N., Setou M. (2009). Layer-specific sulfatide localization in rat hippocampus middle molecular layer is revealed by nanoparticle-assisted laser desorption/ionization imaging mass spectrometry. Med. Mol. Morphol..

[114] Vanderschaeghe D., Festjens N., Delanghe J., Callewaert N. (2010). Glycome profiling using modern glycomics technology: Technical aspects and applications. Biol. Chem..

[115] Hakomori S.I. (1966). Release of carbohydrates from sphingoglycolipid by osmium-catalyzed periodate oxidation followed by treatment with mild alkali. J. Lipid Res..

[116] Wiegandt H., Bucking H.W. (1970). Carbohydrate components of extraneuronal gangliosides from bovine and human spleen, and bovine kidney. Eur. J. Biochem..

[117] Nagahori N., Abe M., Nishimura S. (2009). Structural and functional glycosphingolipidomics by glycoblotting with an aminooxy-functionalized gold nanoparticle. Biochemistry.

[118] Li Y.T., Chou C.W., Li S.C., Kobayashi U., Ishibashi Y.H., Ito M. (2009). Preparation of homogenous oligosaccharide chains from glycosphingolipids. Glycoconjugate J..

[119] Song X., Lasanajak Y., Xia B., Heimburg-Molinaro J., Rhea J.M., Ju H., Zhao C., Molinaro R.J., Cummings R.D., Smith D.F. (2011). Shotgun glycomics: A microarray strategy for functional glycomics. Nat. Methods.

[120] Wenk M.R. (2005). The emerging field of lipidomics. Nat. Rev. Drug Discov..

[121] Magnani J.L., Smith D.F., Ginsburg V. (1980). Detection of gangliosides that bind cholera toxin: Direct binding of 125I-labeled toxin to thin-layer chromatograms. Anal. Biochem..

[122] Muthing J., Schweppe C.H., Karch H., Friedrich A.W. (2009). Shiga toxins, glycosphingolipid diversity, and endothelial cell injurY. Thromb. Haemost..

[123] Musken A., Souady J., Dreisewerd K., Zhang W., Distler U., Peter-Katalinic J., Miller-Podraza H., Karch H., Muthing J. (2010). Application of thin-layer chromatography/infrared matrix-assisted laser desorption/ionization orthogonal time-of-flight mass spectrometry to structural analysis of bacteria-binding glycosphingolipids selected by affinity detection. Rapid Commun. Mass Spectrom..

[124] Meisen I., Friedrich A.W., Karch H., Witting U., Peter-Katalinic J., Muthing J. (2005). Application of combined high-performance thin-layer chromatography immunostaining and nanoelectrospray ionization quadrupole time-of-flight tandem mass spectrometry to the structural characterization of high- and low-affinity binding ligands of Shiga toxin 1. Rapid Commun. Mass Spectrom..

[125] Lopez P.H., Schnaar R.L. (2006). Determination of glycolipid-protein interaction specificity. Meth. Enzymology.

[126] Sampaio J.L., Gerl M.J., Klose C., Ejsing C.S., Beug H., Simons K., Shevchenko A. (2011). Membrane lipidome of an epithelial cell line. Proc. Natl. Acad. Sci. USA.

[127] Dennis E.A., Deems R.A., Harkewicz R., Quehenberger O., Brown H.A., Milne S.B., Myers D.S., Glass C.K., Hardiman G., Reichart D. (2010). A mouse macrophage lipidome. J. Biol. Chem..

[128] Kalvodova L., Sampaio J.L., Cordo S., Ejsing C.S., Shevchenko A., Simons K. (2009). The lipidomes of vesicular stomatitis virus, semliki forest virus, and the host plasma membrane analyzed by quantitative shotgun mass spectrometry. J. Virol..

[129] Imgrund S., Hartmann D., Farwanah H., Eckhardt M., Sandhoff R., Degen J., Gieselmann V., Sandhoff K., Willecke K. (2009). Adult ceramide synthase 2 (CERS2)-deficient mice exhibit myelin sheath defects, cerebellar degeneration, and hepatocarcinomas. J. Biol. Chem..

[130] Iwabuchi K., Prinetti A., Sonnino S., Mauri L., Kobayashi T., Ishii K., Kaga N., Murayama K., Kurihara H., Nakayama H. (2008). Involvement of very long fatty acid-containing lactosylceramide in lactosylceramide-mediated superoxide generation and migration in neutrophils. Glycoconjugate J..

[131] Xu Y.H., Barnes S., Sun Y., Grabowski G.A. (2010). Multi-system disorders of glycosphingolipid and ganglioside metabolism. J. Lipid Res..

[132] Wolf C., Quinn P.J. (2008). Lipidomics in diagnosis of lipidoses. Subcell. Biochem..

[133] Fauler G., Rechberger G.N., Devrnja D., Erwa W., Plecko B., Kotanko P., Breunig F., Paschke E. (2005). Rapid determination of urinary globotriaosylceramide isoform profiles by electrospray ionization mass spectrometry using stearoyl-d35-globotriaosylceramide as internal standard. Rapid Commun. Mass Spectrom..

[134] Nelson B.C., Roddy T., Araghi S., Wilkens D., Thomas J.J., Zhang K., Sung C.C., Richards S.M. (2004). Globotriaosylceramide isoform profiles in human plasma by liquid chromatography-tandem mass spectrometry. J. Chromatogr. B Analyt. Technol. Biomed. Life Sci..

[135] Roddy T.P., Nelson B.C., Sung C.C., Araghi S., Wilkens D., Zhang X.K., Thomas J.J., Richards S.M. (2005). Liquid chromatography-tandem mass spectrometry quantification of globotriaosylceramide in plasma for long-term monitoring of Fabry patients treated with enzyme replacement therapy. Clin. Chem..

[136] Kitagawa T., Ishige N., Suzuki K., Owada M., Ohashi T., Kobayashi M., Eto Y., Tanaka A., Mills K., Winchester B. (2005). Non-invasive screening method for Fabry disease by measuring globotriaosylceramide in whole urine samples using tandem mass spectrometry. Mol. Genet. Metab..

[137] Kruger R., Bruns K., Grunhage S., Rossmann H., Reinke J., Beck M., Lackner K.J. (2010). Determination of globotriaosylceramide in plasma and urine by mass spectrometry. Clin. Chem. Lab. Med..

[138] Whitfield P.D., Sharp P.C., Johnson D.W., Nelson P., Meikle P.J. (2001). Characterization of urinary sulfatides in metachromatic leukodystrophy using electrospray ionization-tandem mass spectrometry. Mol. Genet. Metab..

[139] Fujiwaki T., Yamaguchi S., Tasaka M., Takayanagi M., Isobe M., Taketomi T. (2004). Evaluation of sphingolipids in vitreous bodies from a patient with Gaucher disease, using delayed extraction matrix-assisted laser desorption ionization time-of-flight mass spectrometry. J. Chromatogr. B Analyt. Technol. Biomed. Life Sci..

[140] Fujiwaki T., Tasaka M., Yamaguchi S.  (2008). Quantitative evaluation of sphingomyelin and glucosylceramide using matrix-assisted laser desorption ionization time-of-flight mass spectrometry with sphingosylphosphorylcholine as an internal standard. Practical application to tissues from patients with Niemann-Pick disease types A and C, and Gaucher disease. J. Chromatogr. B Analyt. Technol. Biomed. Life Sci..

[141] Fuller M., Rozaklis T., Lovejoy M., Zarrinkalam K., Hopwood J.J., Meikle P.J. (2008). Glucosylceramide accumulation is not confined to the lysosome in fibroblasts from patients with Gaucher disease. Mol. Genet. Metab..

[142] Kolter T., Sandhoff K. (2006). Sphingolipid metabolism diseases. Biochimica et Biophysica Acta.

[143] Auray-Blais C., Ntwari A., Clarke J.T.R., Warnock D.G., Oliveira J.P., Young S.P., Millington D.S., Bichet D.G., Sirrs S., West M.L. (2010). How well does urinary lyso-Gb(3) function as a biomarker in Fabry disease?. Clin. Chim. Acta.

[144] Sakuraba H., Togawa T., Kawashima I., Kodama T., Tsukimura T., Suzuki T., Fukushige T., Kanekura T. (2010). Tissue and plasma globotriaosylsphingosine could be a biomarker for assessing enzyme replacement therapy for Fabry disease. Biochem. Biophys. Res. Commun..

[145] Schiller J., Zschornig O., Petkovic M., Muller M., Arnhold J., Arnold K. (2001). Lipid analysis of human HDL and LDL by MALDI-TOF mass spectrometry and (31)P-NMR. J. Lipid Res..

[146] Kolter T. (2011). A view on sphingolipids and disease. Chem. Phys. Lipids.

[147] Hernandez-Corbacho M.J., Jenkins R.W., Clarke C.J., Hannun Y.A., Obeid L.M., Snider A.J., Siskind L.J. (2011). Accumulation of long-chain glycosphingolipids during aging is prevented by caloric restriction. PLoS One.

[148] Ariga T., McDonald M.P., Yu R.K. (2008). Role of ganglioside metabolism in the pathogenesis of Alzheimer's disease--a review. J. Lipid Res..

[149] Yuyama K., Yanagisawa K. (2009). Late endocytic dysfunction as a putative cause of amyloid fibril formation in Alzheimer's disease. J. Neurochem..

[150] Han X. (2010). Multi-dimensional mass spectrometry-based shotgun lipidomics and the altered lipids at the mild cognitive impairment stage of Alzheimer's disease. Biochim. Biophys. Acta.

[151] Kabayama K., Sato T., Saito K., Loberto N., Prinetti A., Sonnino S., Kinjo M., Igarashi Y., Inokuchi J. (2007). Dissociation of the insulin receptor and caveolin-1 complex by ganglioside GM3 in the state of insulin resistance. Proc. Natl. Acad. Sci. USA.

[152] Sato T., Nihei Y., Nagafuku M., Tagami S., Chin R., Kawamura M., Miyazaki S., Suzuki M., Sugahara S.I., Takahashi Y. (2008). Circulating levels of ganglioside GM3 in metabolic syndrome: A pilot study. Obes. Res. Clin. Pract..

[153] Inokuchi J. (2011). Physiopathological function of hematoside (GM3 ganglioside). Proc. Jpn. Acad. Ser. B.

[154] Jennemann R., Rothermel U., Wang S., Sandhoff R., Kaden S., Out R., van Berkel T.J., Aerts J.M., Ghauharali K., Sticht C. (2010). Hepatic glycosphingolipid deficiency and liver function in mice. Hepatology.

[155] Fox T.E., Bewley M.C., Unrath K.A., Pedersen M.M., Anderson R.E., Jung D.Y., Jefferson L.S., Kim J.K., Bronson S.K., Flanagan J.M. (2011). Circulating sphingolipid biomarkers in models of type 1 diabetes. J. Lipid Res..

[156] Ryland L.K., Fox T.E., Liu X., Loughran T.P., Kester M. (2011). Dysregulation of sphingolipid metabolism in cancer. Cancer Biol. Ther..

[157] Patwardhan G.A., Liu Y.Y. (2011). Sphingolipids and expression regulation of genes in cancer. Prog. Lipid Res..

[158] Fernandez L.E., Gabri M.R., Guthmann M.D., Gomez R.E., Gold S., Fainboim L., Gomez D.E., Alonso D.F. (2010). NGcGM3 ganglioside: A privileged target for cancer vaccines. Clin. Dev. Immunol..

[159] Miyagi T. (2008). Aberrant expression of sialidase and cancer progression. Proc. Jpn. Acad. Ser. B.

[160] Bernstein M.L. (2011). Targeted therapy in pediatric and adolescent oncology. Cancer.

[161] Yu A.L., Gilman A.L., Ozkaynak M.F., London W.B., Kreissman S.G., Chen H.X., Smith M., Anderson B., Villablanca J.G., Matthay K.K. (2010). Anti-GD2 antibody with GM-CSF, interleukin-2, and isotretinoin for neuroblastoma. N. Engl. J. Med..

[162] Distler U., Souady J., Hulsewig M., Drmic-Hofman I., Haier J., Friedrich A.W., Karch H., Senninger N., Dreisewerd K., Berkenkamp S. (2009). Shiga toxin receptor Gb3Cer/CD77: Tumor-association and promising therapeutic target in pancreas and colon cancer. PLoS One.

[163] Gehrmann M., Liebisch G., Schmitz G., Anderson R., Steinem C., De Maio A., Pockley G., Multhoff G. (2008). Tumor-specific Hsp70 plasma membrane localization is enabled by the glycosphingolipid Gb3. PLoS One.

[164] Zamfir A.D., Serb A., Vukeli Z., Flangea C., Schiopu C., Fabris D., Kalanj-Bognar S., Capitan F., Sisu E. (2011). Assessment of the Molecular Expression and Structure of Gangliosides in Brain Metastasis of Lung Adenocarcinoma by an Advanced Approach Based on Fully Automated Chip-Nanoelectrospray Mass Spectrometry. J. Am. Soc. Mass Spectrom..

[165] Momin A.A., Park H., Portz B.J., Haynes C.A., Shaner R.L., Kelly S.L., Jordan I.K., Merrill A.H. (2011). A method for visualization of "omic" datasets for sphingolipid metabolism to predict potentially interesting differences. J. Lipid Res..

[166] Liu Y., Chen Y., Momin A., Shaner R., Wang E., Bowen N.J., Matyunina L.V., Walker L.D., McDonald J.F., Sullards M.C. (2010). Elevation of sulfatides in ovarian cancer: An integrated transcriptomic and lipidomic analysis including tissue-imaging mass spectrometry. Mol. Cancer.

